# Increased blood pressure in adult offspring of families with Balkan Endemic Nephropathy: a prospective study

**DOI:** 10.1186/1471-2369-7-12

**Published:** 2006-08-23

**Authors:** Plamen S Dimitrov, Valeri A Simeonov, Svetlana D Tsolova, Angel G Bonev, Rossitza B Georgieva, Wilfried J Karmaus

**Affiliations:** 1 National Center of Public Health Protection, 15 "Acad. Ivan Geshov" Street, Sofia, Bulgaria; 2 Vratza District Hospital, "2 June" Street, Vratza, Bulgaria; 3 Department of Epidemiology and Biostatistics, University of South Carolina, 800 Sumter Street, Columbia, South Carolina, USA

## Abstract

**Background:**

Previous studies have linked smaller kidney dimensions to increased blood pressure. However, patients with Balkan Endemic Nephropathy (BEN), whose kidneys shrink during the course of the disease, do not manifest increased blood pressure. The authors evaluated the relationship between kidney cortex width, kidney length, and blood pressure in the offspring of BEN patients and controls.

**Methods:**

102 offspring of BEN patients and 99 control offspring of non-BEN hospital patients in the Vratza District, Bulgaria, were enrolled in a prospective study and examined twice (2003/04 and 2004/05). Kidney dimensions were determined using ultrasound, blood pressure was measured, and medical information was collected. The parental disease of BEN was categorized into three groups: mother, father, or both parents. Repeated measurements were analyzed with mixed regression models.

**Results:**

In all participants, a decrease in minimal kidney cortex width of 1 mm was related to an increase in systolic blood pressure of 1.4 mm Hg (p = 0.005). There was no association between kidney length and blood pressure. A maternal history of BEN was associated with an increase in systolic blood pressure of 6.7 mm Hg (p = 0.03); paternal BEN, +3.2 mm Hg (p = 0.35); or both parents affected, +9.9 mm Hg (p = 0.002). There was a similar relation of kidney cortex width and parental history of BEN with pulse pressure; however, no association with diastolic blood pressure was found.

**Conclusion:**

In BEN and control offspring, a smaller kidney cortex width predisposed to higher blood pressure. Unexpectedly, a maternal history of BEN was associated with average increased systolic blood pressure in offspring.

## Background

There is a large body of evidence that patients with Balkan Endemic Nephropathy (BEN), in contrast to other chronic kidney diseases [1,2], do not manifest increased blood pressure [3-9]. However, little is known about the development of blood pressure in BEN patients, in particular, whether increased blood pressure occurs in preclinical stages. BEN was first characterized in the Vratza District, Bulgaria in 1956 [10]. Later, similar nephropathies were described in Yugoslavia in 1957 [3,10] and in Romania in 1961 [11]. As a result, in 1964, the disease was recognized as a new nosological entity and was named Balkan Endemic Nephropathy. BEN is a tubulo-interstitial kidney disease that progresses slowly for many years. The final disease stage is characterized by renal failure and shrinkage of both kidneys to 3–4 cm in length [12]. Recent studies have shown that the offspring of BEN patients have smaller kidney lengths or cortex widths [13,14], which may be a marker of a reduced number of nephrons [15,16]. Mackenzie et al. advanced the hypothesis that the total nephron number is a factor determining susceptibility to increased blood pressure [17]. In addition, there is evidence that the number of nephrons co-determines kidney dimensions [18]. Lastly, Singh et al. and Zumrutdal et al. have shown that decreased kidney dimensions are associated with increased systolic blood pressure [16,19].

We conducted a prospective study in the Vratza District of Bulgaria, a rural area, and measured the kidney cortex width, kidney length, and blood pressure of the offspring of BEN patients and control offspring at two time points, one year apart. We hypothesize the following:

- A smaller kidney cortex width is related to a higher average systolic blood pressure and a higher pulse pressure.

- An offspring with parental history of BEN has a higher average systolic blood pressure and a higher pulse pressure.

## Methods

### Population

In the period of October, 2003, to April, 2004, we recruited adult offspring (102 study subjects) whose father and/or mother were included in the Vratza Hospital registry of BEN patients in 2001 and who resided in one of three communities (Vratza, Bistretz, or Beli Izvor, Bulgaria). The diagnosis of BEN in the parent generation is based on three groups of criteria: epidemiological, clinical/laboratory, and pathological/anatomical [20]. Regarding epidemiology, the region is known to have a higher prevalence of BEN, thus, the only individual criterion is onset after the age of 20. Clinical and laboratory criteria are a follows: obscure onset, chronic course, no sense of edema, and normochromic type of anemia in more advanced phases. Pathologically, an almost symmetrical macroscopic shrinkage of the two kidneys was required in the parent BEN population. A control group of equal size comprised of adult offspring (99 study subjects) of non-BEN hospitalized patients was enrolled in the study during the same time period. Diagnoses in control parents included diabetes mellitus, cardiovascular disorders, and liver problems. Only three of the 99 parents had kidney disorders (one paternal kidney cancer not related to BEN and two maternal pyelonephritis cases). Subjects of both groups were matched according to gender and ten-year age groups. All participants provided written or verbal consent (witnessed) through a procedure approved by the Institutional Review Board (human-subject research committee) of the National Center of Public Health Protection, Sofia, Bulgaria. The population was enrolled and examined in 2003/04 and investigated again in 2004/05, which provided us with two repeated measurements one year apart.

### Interviews

We conducted face-to-face interviews with all participants either in the hospital, or by visiting them in their home villages. The standardized questionnaire asked for the place of living, type of water supply, diet, smoking and drinking habits, medical symptoms, family history of BEN, family history of other kidney diseases and kidney tumors, and occupational history.

### Physical examination

The physical examination was performed by an experienced physician with board certification in internal diseases and nephrology. It was aimed at assessing the general health status of the study subjects and at revealing symptoms of BEN and/or other internal diseases. Blood pressure was measured according to standards set by the World Health Organization [21]. Participants were in the seated position; three measurements were taken on the right arm at 5 minute intervals. We recorded systolic and diastolic blood pressure and calculated pulse pressure as the difference between these two values.

### Determination of the kidney sizes

Ultrasound investigations of both kidneys took 20–30 minutes. The patient was investigated lying on both the left and right sides. After finding a suitable image, measurements were taken. The longest dimension of the kidney was determined. The thickness of the kidney parenchyma (in the thinnest or minimal part), parenchyma structure, and the relationship of parenchyma and pyelon were measured. Information gathered also included the location, size, and morphology (cysts, stones, and tumors) of the kidneys. The images were saved electronically for future reference. The ultrasonographer (AGB) worked in the Department of Image Diagnostics, Vratza District Hospital, and was blinded to the clinical status of the participant (BEN or control offspring).

### Blood collection and analysis of lead

Blood samples were drawn in K_2_EDTA vacutainers. Lead in blood was determined by using atomic absorption spectrometry. A Model 4110 ZL atomic absorption spectrometer (Bodenseewerk Perkin-Elmer, Ueberlingen, Germany) with a transverse heated graphite atomizer and longitudinal Zeeman-effect background correction equipped with an AS-72 autosampler, electrodeless discharge lamps System II, software WinLab (Version 1.2) and both "Standard"(Part No B 300-0643) and "End-capped" (Part No B 300-0644) THGA tubes with integrated platforms was used for direct electrothermal AAS measurements. To measure lead, blood was diluted 10-fold with 0.25% v/v Triton X-100; 10 μL of the sample and 25 μg of (NH_4_)_2_HPO_4 _as a chemical modifier were injected in the THGA, pre-treated with 250 μg of zirconium and 20 μg iridium. Matrix-matched calibrations were applied [22]. Certified reference materials (Seronorm Trace Elements Whole Blood, Level 1, Cat. No 201505, and Level 2, Cat. No 201605, Sero AS, Norway) were used for internal laboratory control.

### Statistical analyses

Outcome variables were diastolic, systolic blood pressure, and pulse pressure. For descriptive purposes, we defined moderate hypertension as a systolic blood pressure ≥150 mm Hg and a diastolic blood pressure ≥100 mm Hg. However to investigate average increases, the explanatory models used the three continuous outcome variables.

To answer the question whether minimal kidney cortex width is related to higher blood pressure, we controlled for potentially confounding factors such as gender, age, history of smoking, diabetes mellitus, parental history of hypertension or of Balkan Endemic Nephropathy, body surface area, and blood lead level. For smoking, we categorized a subject as being a current smoker, ex-smoker, or non-smoker. A parental history of high blood pressure fell into one of three groups: mother, father, or both affected. We asked for presence of diabetes mellitus in the first investigation (2003/04) and for a new occurrence in the second investigation. The body surface area (BSA) was calculated as ([height * weight]/3600)^2^. This variable is used in the model to control for the effect of weight and height on kidney size and on blood pressure. In each investigation (2003/04 and 2004/05) the average kidney cortex width and kidney length of both kidneys were used as predictors.

To test the hypothesis that offspring of BEN parents have increased blood pressure, we grouped parental disease status into three groups: mother, father, or both parents affected. None of the comparison offspring fell into any of these groups (reference). The effect of cortex width and parental BEN status was tested in the same model.

In order to estimate the association of minimal kidney width and parental history of BEN with blood pressure based on repeated measurements, we applied linear mixed models. Measurements of the two investigations are not independent and mixed models allow adjusting for within-participant effects [23]. The mixed model assumes that the random effects and the error vector are normally distributed, which was the case for the blood pressure variables. SAS PROC MIXED was used to perform the regression analysis [24]. We used Akaike information criteria and the likelihood ratio test to examine the significance of serial correlation (in repeated statement) as well as to model random effects, along with a suitable variance-covariance matrix structure.

To compare the agreement between measurements of continuous variables in 2003/04 and 2004/05 we used the intra-class correlation coefficient (ICC). This is the between-subject minus the within-subject variance divided by the sum of the two variances. The ICC quantifies the proportion of total outcome variance that is due to inter-individual variation.

## Results

Of the 201 participants in the first investigations (2003/04), 189 participated in the follow-up (2004/05, 94%). Two were deceased, one moved out of the area, two could not be contacted, and 7 decided not to participate further. Of the 12 lost, 9 were offspring of BEN patients. Potential risk factors show similar distributions in each of the two investigations (Table 1). One participant had newly developed diabetes. Comparison offspring were about 1.5 to 2 cm taller than BEN offspring; however, the latter were on average 1.5 kg heavier. Blood pressure seems to be higher in BEN offspring: 30.4% were classified as having moderate hypertension (systolic blood pressure ≥140 and/or diastolic ≥90 mm Hg) in the investigation in 2003/04 and 33.3% in 2004/05 (Table 1). The prevalence of moderate hypertension in our rural sample, with a mean age of 50 years, is only marginally lower than the proportion reported in another study of urban population of the same age in Bulgaria [25]. Also, offspring of BEN patients reported more antihypertensive treatment in the year before the investigation.

**Table 1:** Characteristics of the Study Population of the Adult Offspring Study Cohort

		Investigation in 2003/04		Investigation in 2004/05	
		Offspring of BEN patients, n = 102 (%)	Offspring of control patients, n = 99 (%)	Offspring of BEN patients, n = 93 (%)	Offspring of control patients, n = 96 (%)
Gender	male	50.0	47.5	48.8	47.9
Smoking	Current smoker	39.2	34.3	38.7	35.4
	Ex-smoker	22.6	12.1	21.5	12.5
	Non-smoker	38.4	53.5	39.8	52.1
Diabetes	Yes	7.8	6.1	5.4	7.3
Parental history of hypertension	Mother	19.6	16.2	20.4	16.7
	Father	13.7	16.2	15.1	16.7
	Both	2.9	5.1	3.2	5.2
Parental history of BEN	Mother	38.2	0	38.7	0
	Father	25.2	0	25.8	0
	Both	36.3	0	35.5	0
Moderate hypertension in the offspring^ξ^		30.4	19.2	32.3	22.9
Antihypertensive medication		22.5	5.1	29.0	12.5

		Mean (standard deviation) or median and 95% confidence interval^†^			
Age	(years)	47.3 (9.4)	49.6 (9.1)	50.4 (9.2)	48.8 (9.6)
Systolic blood pressure	(mm Hg)	130.7 (20.1)	124.9 (17.6)	135.1 (23.0)	126.1 (19.1)
Diastolic blood pressure	(mm Hg)	80.8 (12.2)	78.9 (10.5)	83.5 (14.0)	80.6 (12.5)
Pulse pressure ^$^	(mm Hg)	49.3 (13.3)	46.2 (12.1)	51.5 (13.3)	45.5 (10.7)
Weight	(kg)	75.8 (14.8)	73.6 (11.6)	73.3 (14.2)	71.9 (11.1)
Height	(cm)	167.7 (8.5)	169.4 (7.7)	163.8 (9.0)	165.3 (7.7)
Body surface area ^#^	(m^2^)	1.87 (0.22)	1.86 (0.2)	1.82 (0.21)	1.81 (0.2)
Minimal width of the kidney cortex	(mm)	15.5 (2.03)	15.9 (1.9)	15.2 (2.1)	15.6 (1.8)
Kidney length	(mm)	116.7 (6.8)	119.6 (7.0)	117.3 (6.8)	119.2 (6.8)
Lead in whole blood	(μg/L, median and 95% CI)¥	93.8 (50–205)	87.6 (42–225)	87.7 (41–210)	84 (39–203)

† A median was used if the variable was not normally distributed.

$ Difference of systolic minus diastolic blood pressure

¥ 95%CI – 95% confidence interval

# Body surface area = ([height * weight]/3600)2

ξ Systolic blood pressure ≥140 and/or diastolic ≥90 mm Hg.

**Figure 1:**
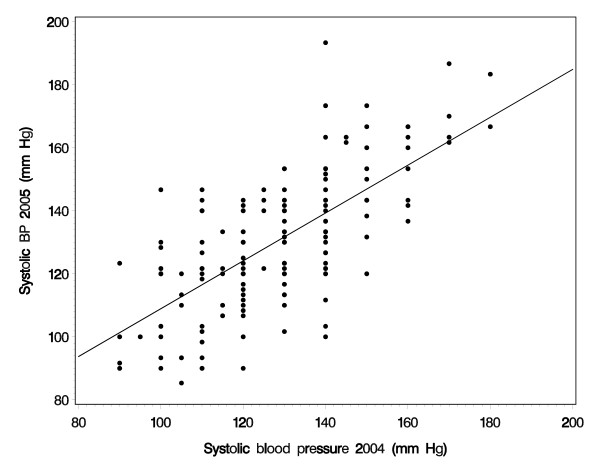
Comparisons of systolic blood pressure measurements in 2003/04 and 2004/05.

Figure 1 shows the agreement between the two systolic blood pressure measurements one year apart. The intraclass correlation coefficient (ICC) of 0.68 (lower 5% limit: 0.61) was good for systolic blood pressure, but lower for diastolic blood pressure (ICC = 0.49, lower 5% limit: 0.39). For the minimal cortex width, the ICC was 0.66 (lower 5% limit: 0.58) and 0.92 for kidney length (lower limit: 0.89). Blood lead concentration also showed good agreement (ICC = 0.65, lower limit: 0.57).

The study population consists of 201 subjects that provided 388 observations (two measurements for most participants, one year apart). When analyzing blood pressure differences while controlling for confounders, cortex width and parental BEN status had significant effects on systolic and pulse pressure, but not on diastolic blood pressure. A decrease in the minimal kidney cortex of 1 mm was linearly related to an increase in systolic blood pressure of 1.4 mm Hg (p = 0.005) and an increase of pulse pressure of 1.1 mm Hg (p = 0.002).

**Figure 2:**
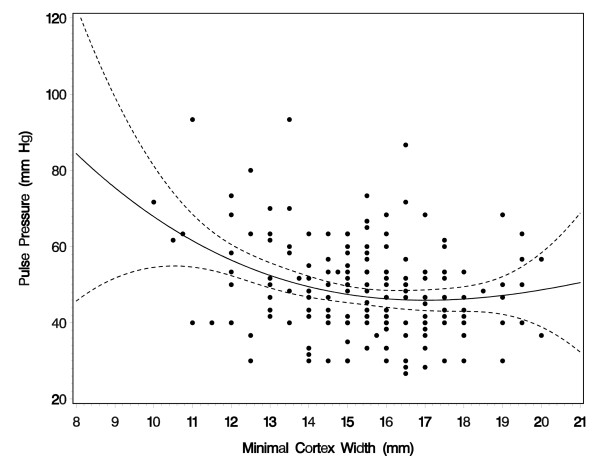
Scatterplot of minimal kidney cortex width with pulse pressure in the 2004/05 investigation (crude association; for adjusted effects, see Table 2). The straight line shows the estimated cubic association, the dotted lines show the 95% confidence limits.

Figure 2 shows the scatter plot of minimal cortex width and pulse pressure using a cubic regression with 95% confidence limits. A cubic regression fitted the observations best when no other predictors were taken into consideration. Within the boundaries of our measurements of the cortex width there was no decrease in blood pressure once the minimal kidney cortex width was 16 mm or larger. Blood pressure started to increase when the width was smaller than 16 mm.

A maternal history of BEN was associated with an increased systolic blood pressure in offspring of 6.7 mm Hg (p = 0.03), and when both parents had BEN, an increased systolic blood pressure of 9.9 mm Hg (p = 0.002). There was no statistically significant effect of a paternal history of BEN.

To investigate whether the use of antihypertensive medication had distorted our findings, we repeated the model excluding those observations with antihypertensive drug use. This reduced the number of repeated observation from 388 to 322 (176 subjects), but did not change our findings.

## Discussion

Our findings show that a decrease of the minimal kidney cortex width of 1 mm was related to an increase in systolic blood pressure of 1.4 mm Hg. There was no association between kidney length and blood pressure. A maternal history of BEN was associated with a significant increase in systolic blood pressure of 6.7 mm Hg, paternal BEN with a non-significant increase of 3.2 mm Hg, and both parents affected, a significant increase of 9.9 mm Hg. The lack of association of either kidney cortex width or a parental history of BEN with diastolic blood pressure may either stem from the larger individual variance of this parameter or may indicate that there is no such link.

In a study with healthy volunteers in England, Raman et al. did not identify a clear pattern between measurements of the right and left kidneys and blood pressure [26]. In another population-based study, Paivansalo et al. found that the kidneys of hypertensive women were slightly larger (p < 0.08) [27]. To the contrary, comparing hypertensive and normotensive Turkish patients, Zumrutdal et al. found that kidney length and volume were significantly smaller in hypertensive patients [19]. Thus, it is not clear whether hypertension affects kidney dimensions or vice versa.

For adult Aboriginals in Australia, Singh et al. found that a 10 mL decrease in kidney volume was associated with a 0.5 mm Hg increase in systolic blood pressure [16]. The study of Singh et al. did not provide information on cortex width; in our study we do not have information on kidney volume. To compare results, we expressed both findings as a percent decrease in kidney dimensions. A 10 mL decrease in kidney volume in the Australian study represents approximately a 5% decrease in volume (range: 62 – 267 mL). In our study of adult Caucasian offspring from Bulgaria, a 5% decrease in the minimal cortex width (0.5 mm, range 10 – 20 mm) is associated with a 0.7 mm Hg increase in blood pressure. Thus, both studies found a similar effect of kidney dimensions on blood pressure. Singh et al. also showed a cubic-type association between kidney volume and systolic blood pressure. Similarly, we have demonstrated a cubic regression between minimal kidney cortex width and blood pressure; the threshold we detected in our model, is approximately 16 mm of cortex width. However, if we control for other variables in mixed regression models, a straight line is also appropriate (Table 2).

**Table 2:** Effect of kidney Measures and Parental History of Balkan Endemic Nephropathy on Blood Pressure in Adult Offspring

		Diastolic blood pressure		Systolic blood pressure		Pulse pressure	
		Parameter estimate^# ^(mm Hg)	p-value	Parameter estimate^# ^(mm Hg)	p-value	Parameter estimate^# ^(mm Hg)	p-value
Minimal width of the kidney cortex	(mm)	-0.6	0.07	-1.5	0.005	-1.1	0.002
Kidney length	(mm)	0.1	0.29	0.2	0.42	0.0	0.80
Parental history of BEN	Mother	2.0	0.25	6.7	0.03	4.3	0.02
	Father	1.9	0.33	3.2	0.35	1.5	0.48
	Both	3.3	0.06	9.9	0.002	6.3	0.001

# Adjusted for sex, age, body surface area, diabetes, parental history of hypertension (mother, father, both, none), smoking (current, ex-smoker, never), and lead blood concentration.

Mackenzie et al. posited the hypothesis that the total nephron supply at birth is a factor in determining a susceptibility to increased blood pressure [17]. Given that kidney dimensions correlate with the number of nephrons [18], our finding that minimal cortex width is significantly associated with systolic and pulse pressure supports Mackenzie's hypothesis. In addition, we found that, in offspring, maternal BEN is associated with increased systolic and pulse pressure. In a prior report of this project, we showed that a maternal but not a paternal history of BEN is related to reduced cortex width in their adult offspring [14]. The stronger role of maternal BEN in offspring susceptibilities (cortex width and average blood pressure) and the lack of paternal-related risks may indicate, in our opinion, the importance of feto-maternal interactions during pregnancy. Mothers who later develop BEN do not yet have the disease during their childbearing years. However, they are likely to have developed precursors of the disease before becoming pregnant, which, however, must be sufficient in themselves to initiate a higher risk in the offspring for having a smaller kidney cortex width and increased systolic and pulse pressure.

Hence, a maternal history of BEN may have both a direct and an indirect effect on blood pressure in offspring. Indirectly, maternal BEN seems to decrease kidney cortex width, which in turn increases blood pressure in adult offspring. Directly, maternal BEN seems to initiate regulations likely to raise the average blood pressure in affected offspring. However, hypertension is not a predominant feature in BEN offspring. Thus, it is unlikely that the reduction in kidney cortex width in BEN offspring results from hypertension.

Renal mass is considered to contribute directly to the development and maintenance of hypertension [16,19,28]. Also renal scarring, an index of chronic damage, has also been related to blood pressure [29]. Since we have previously reported a reduced kidney cortex width in BEN offspring [14], our finding that minimal cortex width is related to blood pressure is in agreement with prior reports.

However, in BEN patients, whose kidneys shrink, the lack of increased blood pressure has been considered to be a typical characteristic before the disease reaches its final stage [3-9]. The reason for this unusual association has never been explained. We were surprised to find that BEN offspring had an average increased systolic blood pressure, in particular related to a maternal history of BEN. In agreement with our finding, Arsenovic et al. recently also reported an increased prevalence of hypertension (20 out of 47 persons) in BEN family members [30].

Since our data is based on adult offspring, we are uncertain about the future development of blood pressure in this group. There are three possibilities: (I) Offspring of BEN patients will not develop BEN but maternal BEN may lead to subclinical changes such as an increase in average blood pressure. An argument against this option is that we have found reduced kidney sizes and increased protein excretion in the BEN offspring, which may be considered as early signs of BEN [14]. (II) BEN offspring will develop BEN and their higher average blood pressure will disappear with the progression of BEN. One speculation is that the juxtaglomerular cells become injured and thus the renin-angiotensin system also is impared, which may prevent hypertension. (III) A third option is that the reported lack of hypertension in BEN patients was not based on scientifically sound conclusions and needs to be revised.

## Conclusion

We found that adult offspring of BEN patients have an average increased blood systolic pressure and pulse pressure, determined in two repeated measurement one year apart. This is contrary to reports in BEN patients, who do not have this characteristic. Surprisingly, the increase was related to a maternal history of BEN, or a history of BEN in both parents, but not to a paternal history alone. Increased blood pressure was also associated with a reduction of the minimal width of the kidney cortex, but not with a reduction in kidney length. In our understanding, these findings emphasize the role of maternal priming in determining susceptibility for increased blood pressure. Balkan endemic nephropathy may serve as a model to better understand the development of both kidney disorders and their related blood pressures.

## Competing interests

The author(s) declare that they have no competing interests.

## Authors' contributions

PSD developed and designed the study and helped write the report. SDT assisted in analyzing the data, literature review, and writing the discussion. RBG conducted the analyses of lead in blood, and assisted in writing the discussion. VAS conducted the clinical examinations of the study subjects and contributed to the interpretation. AGB conducted the ultrasound examinations of the study subjects and contributed to the interpretation. WJK helped design the study and conducted the statistical analysis and worked on the manuscript. All authors read and approved the final manuscript.

## Pre-publication history

The pre-publication history for this paper can be accessed here:


